# Anterior Impingement Syndrome of the Ankle Caused by Osteoid Osteoma in the Talar Neck Treated with Arthroscopy and 3D C-Arm-Based Imaging

**DOI:** 10.1155/2017/2171627

**Published:** 2017-03-28

**Authors:** Masachika Ikegami, Takumi Matsumoto, Song Ho Chang, Hiroshi Kobayashi, Yusuke Shinoda, Sakae Tanaka

**Affiliations:** Department of Orthopaedic Surgery, Faculty of Medicine, The University of Tokyo, 7-3-1 Hongo, Bunkyo-ku, Tokyo 113-0033, Japan

## Abstract

Osteoid osteoma in periarticular lesions tends to have an unusual presentation that likely leads to a delayed or missed diagnosis compared with a typical osteoid osteoma in the metaphysis or diaphysis of the long bone. In cases that are unresponsive to conservative treatment, surgical interventions including en bloc resection, computed tomography-guided percutaneous treatment, and arthroscopic resection have been performed; however, these methods frequently result in inadequate tumor resection and recurrence. Here we present a case of a 16-year-old girl with osteoid osteoma in the talar neck presenting as anterior impingement syndrome due to marked synovitis in the ankle joint which was successfully treated without complications by arthroscopic synovectomy and tumor resection followed by intraoperative 3D C-arm-based imaging confirming complete tumor lesion removal. Her pain was relieved immediately after the surgery, and there was no recurrence at 12 months of follow-up. This is the first case report of the surgical treatment of the osteoid osteoma in the talar neck with the combination methods of arthroscopy and 3D C-arm-based imaging.

## 1. Introduction

Osteoid osteoma (OO) is a common benign osteoblastic bone tumor that affects mainly children and young adults [1]. Although OO can occur in any bone of the human skeleton, the metaphyseal and diaphyseal regions of the femur and tibia are most commonly affected and comprise approximately half of known cases [2]. The talus is involved in 2–10% of OO cases, and 97% of talar OO cases are located in the talar neck [3]. Periarticular positioning of the OO in the talar neck makes the correct diagnosis difficult due to the unusual presentation mimicking monoarthropathy and the obscure radiographic findings with less periosteal response than typical intracortical lesions in the long bone. OO in the talar neck could be misdiagnosed as other conditions accompanied by chronic ankle pain including ankle impingement syndrome, stress fracture, osteonecrosis, osteomyelitis, chronic ankle sprain, and inflammatory arthropathy. The average lag time between symptom onset and a diagnosis of OO in the talus is reportedly 2-3 years [4, 5]. En bloc resection and thermal destruction with laser or radiofrequency ablation under radiological guidance are the most common surgical treatments for OO in the talus [5], although management attempts using arthroscopy were also recently reported [6]. Arthroscopy is technically demanding but has the advantage of being less invasive than arthrotomy; however, the risk of recurrence due to inadequate excision under a limited surgical field of view into the nidus via a small window of the cortex is the greatest concern [7]. Here we report an unusual case of OO in the talar neck presenting as anterior ankle impingement syndrome in a 16-year-old girl. We then report the use of intraoperative 3D C-arm-based imaging and discuss its usefulness as an arthroscopic procedural aid.

## 2. Case Report

A 16-year-old Japanese girl with no past medical history or previous injuries presented to a nearby clinic complaining of chronic anterior left ankle pain for the past 2 years. She was diagnosed with anterior ankle impingement syndrome by ultrasonographic findings of synovial hyperplasia in the anterior aspect of the ankle joint and referred to our hospital for further treatment. A physical examination revealed tenderness and swelling across the anterior aspect of the left ankle but no local heat or redness of the overlying skin. She played badminton three times a week and complained of increasing pain with sports activities. Careful interviewing revealed that she also had pain at rest which increased with motion in the morning which was strong especially immediately after awakening.

**Figure 1:**
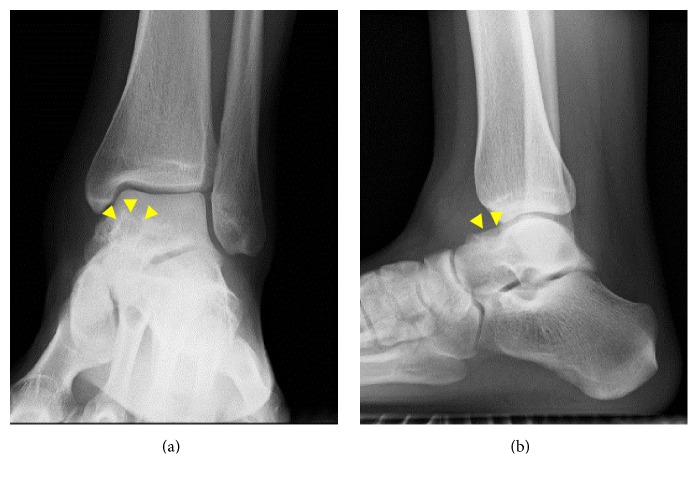
Anteroposterior (a) and lateral (b) plain radiographs of the left ankle showing a radiolucent lesion with marginal sclerosis in the talar neck (arrow-heads).

**Figure 2:**
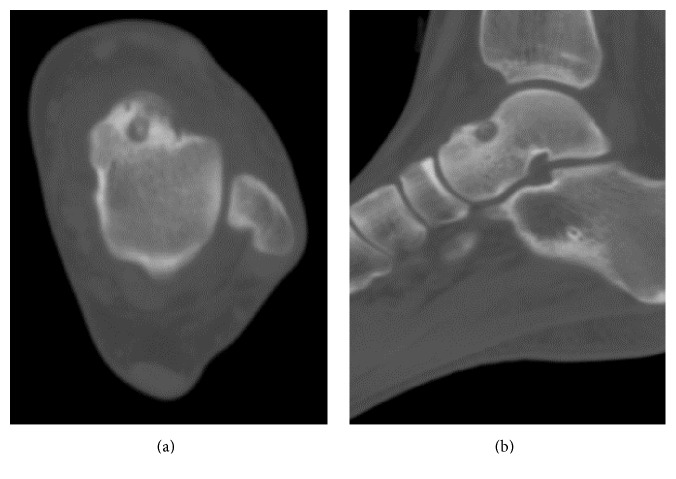
Axial (a) and sagittal (b) computed tomography images of the left ankle showing central calcification within a radiolucent lesion of the talar neck.

**Figure 3:**
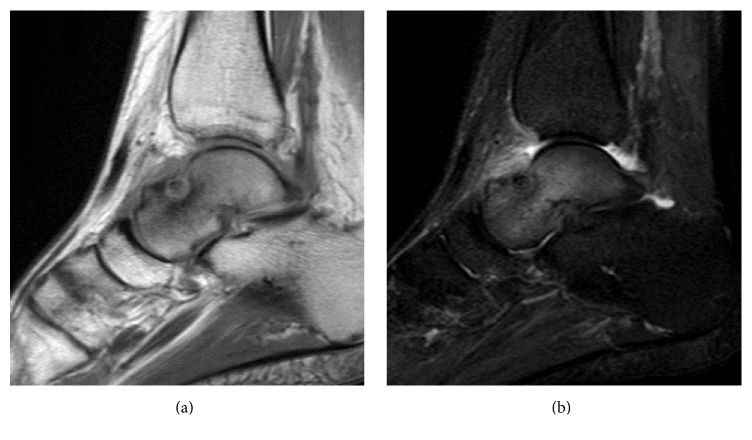
T1-weighted (a) and short T1 inversion recovery (b) sagittal magnetic resonance imaging showing a bone lesion in the talar neck with surrounding bone marrow edema and synovitis in front of the bone lesion.

The range of motion of the left ankle was normal, and marked pain was observed with forced dorsiflexion. There were no signs of ankle instability. A blood test showed that her white blood cell count, C-reactive protein level, and matrix metalloproteinase-3 level were within normal limits. Rheumatoid factor and anticyclic citrullinated peptide antibody tests were negative. Plain radiographs showed a small exostotic bony bulge on the talar neck which resembled a traction spur and a recess on the talar neck (Figure 1). Computed tomography (CT) showed an 8 mm radiolucent lesion with marginal sclerosis and central calcification in the talar neck (Figure 2). Magnetic resonance imaging (MRI) revealed a bone lesion in the talar neck with surrounding bone marrow edema, synovial thickening in front of the bone lesion, and joint effusion (Figure 3).

We initially considered two pathologies as differential diagnoses: one was anterior ankle impingement syndrome considering the bone lesion as a recess, flake, and spur caused by impaction of the distal tibia against the talar neck; and the other was OO in the talar neck considering the bone lesion in CT and MRI as a nidus with secondary synovitis in the ankle joint. Activity modification and daily oral aspirin therapy slightly reduced but did not eliminate her symptoms. An intra-articular steroid injection provided some pain relief; however, the effect lasted only 1 week. Persistent synovitis unresponsive to conservative treatments for several months prompted us to narrow down the differential diagnosis to OO. The patient opted for conservative treatment consisting of oral aspirin or nonsteroidal anti-inflammatory drugs (NSAIDs); however, her symptoms persisted and she finally decided to undergo surgical resection 14 months after the first visit.

**Figure 4:**
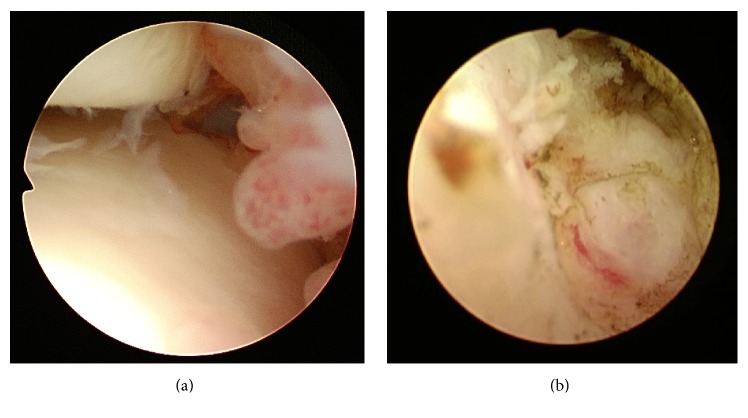
(a) Arthroscopy of the left ankle joint. Hyperplasia with hyperemia of the joint synovium was noted. (b) After synovectomy, a red subperiosteal lesion was seen through the thinned cortex of the talar neck.

**Figure 5:**
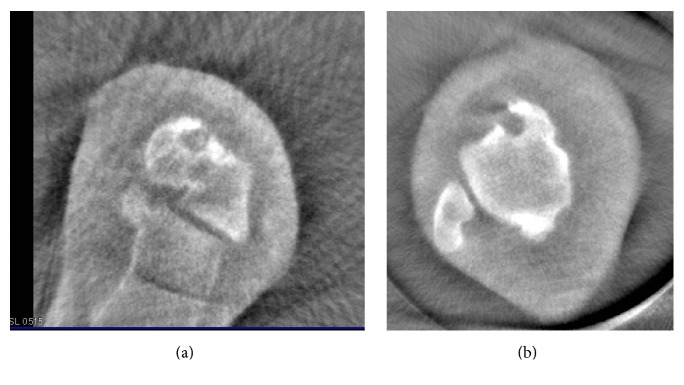
Intraoperative 3D C-arm-based axial reconstructed imaging before (a) and after (b) tumor resection. (a) Precise detection of the nidus was achieved before destruction of the cortex. (b) Complete tumor resection and appropriate bone preservation were confirmed intraoperatively.

We performed arthroscopic surgery using anteromedial and anterolateral portals. The patient was placed in the supine position on a radiolucent carbon fiber table with the ankle manually distracted. Arthroscopy revealed capillary hyperemia and synovial hyperplasia in the anterior aspect of the ankle joint (Figure 4(a)). We performed a thorough synovectomy with a shaver and radiofrequency probe to obtain a clear and larger operative field. The surface of the OO lesion was suspected after exposure because the overlying cortex was irregular and too thin to exhibit evidence of denting under gentle pressure with a probe (Figure 4(b)). We then used 3D C-arm-based imaging (ARCADIS Orbic 3D, Siemens Medical Solutions, Erlangen, Germany) to verify the lesion's exact location and visualize the exact extent of the nidus without removal of large part of cortex (Figure 5(a)) and used grasping forceps and curettes to remove it. The entire shell of the lesion could not be visualized arthroscopically after thorough resection; therefore, we checked the lesion with another 3D scan. Unexpectedly, a remnant lesion was confirmed in a dead angle of the arthroscope. We resected the residual nidus and marginal sclerotic bone using curettes and a radiofrequency probe. A final 3D scan was performed to confirm that the nidus was completely removed (Figure 5(b)). The cavity of the excised tumor was left empty without any augmentation. The excised nidus and obtained synovium were sent separately for histopathological examination. The histopathology of the synovium was consistent with inflammatory synovitis, while the excised nidus showed randomly interconnecting trabeculae of the osteoid in a fibrovascular stroma rimmed by osteoblasts which was consistent with OO.

Postoperatively, the patient reported immediate relief of her ankle pain. She was followed up for 12 months without lesion recurrence. Symptoms of anterior ankle impingement disappeared with no functional disability at the latest follow-up.

## 3. Discussion

OO in the foot typically involves a subperiosteal lesion of the talar neck [3]. The diagnosis of intra-articular OO is often delayed or missed due to its unusual presentation mimicking more common conditions represented by impingement syndrome, and several studies have raised alarm over this kind of misdiagnosis in various joints [8–11]. We also emphasize from our experience in the present case that intra-articular OO can induce soft-tissue impingement syndrome by causing chronic synovitis. It is essential that OO be considered in the differential diagnosis of impingement syndrome, in adolescence particularly. The sensitivity of plain radiographs for diagnosing the intra-/juxta-articular OO of the talar neck is reportedly as low as 61.5% [5]. MRI presentation in an impingement syndrome includes synovitis, joint effusion, and bone marrow effusion of the areas of impact, which is difficult to discriminate from those of intra-articular OO [12]. CT has higher sensitivity than MRI, typically demonstrating a low attenuation nidus with focal central calcification and surrounding sclerosis which could not be shown clearly in MRI [5, 13]. Unlike extra-articular and intracortical cases usually showing strong periosteal reaction, intra-articular OO typically lacks periosteal reaction because of absence of functional periosteum within the joint [13].

Prostaglandins produced in OO via the strong expression of cyclooxygenase-1 (COX-1) and COX-2 are considered to cause characteristic nocturnal pain that is responsive to nonsteroidal anti-inflammatory drugs (NSAIDs) in typical OO and synovitis in OO located within periarticular lesions [14]. The natural course of OO is thought to be spontaneous healing within 6–15 years [15, 16], and NSAIDs reportedly shorten this time to 2-3 years [17]. When the location of OO is difficult to remove or the patient refuses to undergo surgery, the tumor is a good candidate for nonoperative treatment. However, NSAIDs are ineffective in one-third of patients for whom surgical intervention is required [17].

OO is treated by several invasive methods including open curettage, en bloc resection, CT-guided percutaneous local ablation, and arthroscopic resection [5]. Open removal of the OO nidus has been the standard treatment with a success rate of 94.9% [18]. The disadvantage of this surgical technique is that a large bone defect is required to find out the nidus and achieve thorough lesion removal because OO usually resembles the gross surrounding normal bone. Complications including fracture and infection after open surgeries were reported in 7–45.5% of cases [18]. Patients often require partial weight bearing or restricted activity for a few months because of the weakened bone strength.

The open surgical excision of OO was recently replaced by less invasive methods. CT-guided percutaneous therapies including core drill excision [19], laser ablation [20], and radiofrequency ablation [21] can preserve the surrounding normal bone structure by avoiding extensive digging of the cortex to find out the OO nidus. While the lower invasiveness and smaller bone defect of these methods have the advantage of short hospitalization and convalescence periods, they are accompanied by a higher recurrence rate of 13.5% [18]. Another disadvantage of these methods is difficulty obtaining reliable pathological specimens, especially when the bone surrounding the OO is very hard or the lesion is very small. Moreover, since percutaneous methods are often performed outside the operating room, it is difficult to maintain a sterile environment. The overall complication rate of the methods was reportedly up to 24% [18].

Arthroscopic excision is another minimally invasive treatment option for intra-articular OO which has shown good clinical results [6, 22–26]. Unlike using percutaneous methods, tumor resection and synovectomy can be performed. Therefore, cases with chronic synovitis like that described here are good candidates for arthroscopic treatment. As the talar neck can be easily exposed by arthroscopy, 10.9% of reported cases of talar OO were treated by arthroscopic excision [5]. The only case series about arthroscopic procedures included nine cases with good clinical outcomes, no recurrence, and no pain at a mean follow-up of 6 years [6]. However, due to limited surgical field of view and access for surgical devices, recurrence after incomplete resection with arthroscopy has been reported [7]. Another case of completely sclerotic OO in the talar neck which could not be detected and was irremovable arthroscopically has been also reported [27]. As arthroscopic treatment depends greatly on surgeons' skills and experience, it is essential to use the appropriate arthroscopic technique and accurate imaging guidance to avoid an incomplete resection, especially when arthroscopic visualization is not adequate or the surgeons are not confident in the complete removal.

Several studies have reported that minimally invasive surgery of OO with 3D C-arm-based imaging is a highly effective and safe procedure [28–31]. This technique with real-time 3D imaging guidance enables precise intraoperative nidus localization and confirmation of the resection extent, which enable surgeons to achieve complete lesion resection without destroying more bone than necessary [31]. Although a limited number of studies have reported the use of 3D imaging in the treatment of OO, no reports to date have detailed recurrence or complications after this technique [28–31].

Intraoperative CT imaging (O-arm, Medtronic, Minneapolis, USA) is another option for intraoperative imaging. O-arm delivers higher image quality and quicker 3D reconstruction time than 3D C-arm; however, O-arm has some disadvantages [32]. First, its O-shaped design makes it suited mainly to spinal surgeries. Second, O-arm exposes patients to larger doses of radiation. Third, O-arm is costly in terms of purchase and maintenance. On the other hand, 3D C-arm, with a standard C-arm design, has great usability and a broad range of surgical applications with lower running cost. Usefulness of 3D C-arm with or without navigation system in foot and ankle surgeries has been reported in the previous literatures [33, 34]. Exposure to radiation is one of the disadvantages of this technique; however, the exposed dose by 3D C-arm is expected to be comparable to or less than that by a diagnostic CT [35]. For the combination use with ankle arthroscopy, we consider that 3D C-arm is a better modality than O-arm.

To our knowledge, this is the first report to describe a combination method of arthroscopy and 3D C-arm-based imaging. Based on the previous studies and our experience in the present case, we believe that this combination method is a useful option for the treatment of intra-articular OO. The advantages of this method against arthroscopy alone are capabilities to access the nidus easily and less invasively and to confirm the complete resection. The disadvantages are its cost and radiation exposure to patients, though the amount is of acceptable level.

## 4. Conclusions

We described a case of talar neck OO showing an unusual presentation of anterior ankle impingement syndrome. Using arthroscopy and 3D C-arm-based imaging, we performed a synovectomy, clearly exposed the nidus, and intraoperatively confirmed complete lesion removal without complications.
